# Comparison of the measurement properties of EQ-5D-5L and SF-6Dv2 in Chinese patients on dialysis

**DOI:** 10.1186/s12955-025-02403-w

**Published:** 2025-07-10

**Authors:** Ye Zhang, Zeyuan Chen, Li Yang, Johan Jarl

**Affiliations:** 1 Population Development Studies Center, Renmin University of China, Beijing, 100872 People’s Republic of China; 2 School of Population and Health, Renmin University of China, Beijing, 100872 People’s Republic of China; 3 Department of Informatics and Media, Uppsala University, Uppsala, SE-751 05 Sweden; 4 School of Public Health, Peking University, Beijing, 100191 People’s Republic of China; 5 Health Economics Unit, Department of Clinical Sciences (Malmö), Forum Medicum, Lund University, Sölvegatan 19, Lund, 223 62 Sweden

**Keywords:** EQ-5D-5L, SF-6Dv2, Measurement properties, Dialysis

## Abstract

**Background:**

Measuring health-related quality of life (HRQoL) in dialysis patients is essential for clinical assessment and economic evaluation. Despite the emergence and increasing use of updated instruments, evidence comparing their performance in Chinese dialysis patients remains limited. The aim of this study was to compare the measurement properties of the EQ-5D-5L and the SF-6Dv2 instruments in Chinese patients on dialysis and to provide a reference for future utility scale choice for Chinese dialysis patients.

**Methods:**

Data were obtained using Wen Juan Xing questionnaire from dialysis patients during November 2023 to January 2024 in hospitals in China. The questionnaire included the EQ-5D-5L, SF-6Dv2, the kidney disease quality of life instrument (KDQOL-36), clinical and socio-demographic characteristics. The agreement of utility scores was assessed using intra class correlation coefficients (ICC) and Bland-Altman plots. The construct validity was evaluated using Spearman’s correlation coefficient. The known group validity was evaluated by comparing the scores among patients with different health states, and sensitivity was compared using relative efficiency and the effect size.

**Results:**

A total of 378 patients (male, 49.5%; mean age, 49.1 years) were included in this study. The ICC between EQ-5D-5L and SF-6Dv2 utility values was 0.639. The correlation between the two scales was strong (0.767). Both scales showed known groups validity, although the SF-6Dv2 was more sensitive. The differences in the SF-6Dv2 scores for patients in better and worse health state were greater than those measured by the EQ-5D-5L scores.

**Conclusions:**

Both EQ-5D-5L and SF-6Dv2 instruments are valid for dialysis patients. However, the two scales cannot be used interchangeably, and it appears that the SF-6Dv2 was more sensitive in capturing health state differences for dialysis patients in China.

## Introduction

Economic evaluation has been increasingly used in health policy decision making. Quality-adjusted life-year (QALY) has gained higher attention as the primary outcome for economic evaluation. Preference based health-related quality of life (HRQOL) measures are commonly used to generate utility scores for calculating QALYs.

The EQ-5D and the SF-6D are two commonly used preference based HRQOL measures [1, 2]. Both measures can produce health state utility scores using the preference on different health states by the general population and are often assumed to be comparable [3–5]. However, this is not necessarily the case and the results might differ depending on the choice of preference based instrument. It is therefore important to examine possible differences between instruments in order to recognize their impact on the calculation of QALYs and on cost-effectiveness analyses.

Previous studies have explored the performance of EQ-5D and SF-6D across diverse populations, encompassing healthy individuals, patients with late-onset muscular dystrophy, lymphoma, and chronic low back pain [3–6]. However, the results differed between studies. For example, one study found that EQ-5D-5L generated greater utility scores and had stronger discriminative power of health states compared to SF-6Dv2 among patients with pompe disease [4], while another study found that SF-6Dv2 demonstrated superior discriminatory capability in Chinese healthy individuals compared to EQ-5D-5L [3].

The EQ-5D and SF-6D have been used in patients with dialysis and both are considered valid measures [7]– [8]. However, the discriminatory capability of these two instruments, and thus which one is more suitable, remains unknown in a Chinese setting for dialysis patients. In addition, most previous studies utilized the EQ-5D-3L, which is susceptible to ceiling effects and exhibits poor discriminatory capability, with the exception of a study conducted in Singapore that employed the EQ-5D-5L [7, 9, 10]. The latest version of the SF-6D (SF-6Dv2) has also rarely been employed in previous studies [7, 8]. Dialysis patients typically experience complex and fluctuating health states, which require accurate HRQOL measurement. Therefore, the evaluation and comparison of these updated instruments in the dialysis population is particularly relevant. Besides, utility measures like the EQ-5D-5L and SF-6Dv2 rely on self-reported outcomes, which can be affected by local norms, response styles, and value sets used to derive utility scores. It is therefore particularly important to compare these instruments in the region-specific context.

The aim of this study was to compare the measurement properties of EQ-5D-5L and SF-6Dv2 instruments in Chinese patients with end-stage renal disease in terms of agreement, construct validity, and sensitivity.

## Methods

### Study design and patients

The data were collected from a multicenter survey conducted between November 2023 and January 2024 for dialysis patients in China. The questionnaire was distributed through the largest online survey platform in China, Wen Juan Xing (Changsha Ranxing Information Technology Co., Ltd., Hunan, China). Participants were mainly outpatients admitted to eight hospitals in four big cities (Beijing, Xian, Chengdu, and Hangzhou) in China and the participating hospitals were the main nephrology centers of each city. To minimize selection bias due to digital illiteracy, face-to-face interviews were used to help complete the survey for those patients who were unable to use electronic devices. To reach the largest possible number of participants, the survey was also distributed in dialysis patient WeChat groups which have been certified by the relevant nephrologist from included hospitals. With this, we aimed to reach a wider and different sample of patients who have experienced dialysis from hospitals at different levels in different regions of China. The inclusion criteria were (1) diagnosed with end stage renal disease (ESRD), (2) going through dialysis therapies for at least three months, (3) able to communicate normally and independently, (4) agreeing to participate in the study, and (5) given informed consent. The exclusion criteria were patients with severe mental illness or cognitive impairment that unable to participate in the survey. The study was approved by the Ethics Committee for Research at Renmin University of China.

### Data collection

The self-reported questionnaire included sociodemographic characteristics (age, sex, educational level, marital status), clinical data such as the number of comorbidities, and HRQOL measures that include EQ-5D-5L self-report questionnaire, SF-6Dv2 self-report questionnaire, and the 36-item Kidney Disease Quality of Life questionnaire (KDQOL-36).

#### EQ-5D-5L

The EQ-5D-5L questionnaire has a health-state descriptive system and a visual analog scale (EQ-VAS). The descriptive system includes five items: mobility (MO), self-care (SC), usual activities (UA), pain/discomfort (PD), and anxiety/depression (AD). Each item has five descriptive levels which include “no problems’’, ‘‘slight problems’’, ‘‘moderate problems’’, ‘‘severe problems’’, and “unable to do’’ or ‘‘extreme problems’’.

Individuals were asked to rate their health based on “today” as the recall period. Responses to the five EQ-5D-5L items can define 3,125 health states in total. For each health state, an index score can be generated using a value set. In this study, the Chinese EQ-5D-5L value set developed by Luo et al. was used to calculate the EQ-5D-5L index score, ranging from − 0.391 (the worst health state) to 1 (full health) [11, 12].

#### SF-6Dv2

SF-6Dv2 questionnaire includes six dimensions: physical functioning (PF), role limitation (RL), social functioning (SF), pain (PN), mental health (MH), and vitality (VT), with 5–6 levels for each dimension. With a standard 4-week recall period, SF-6Dv2 can measure 18,750 health states in total. In this study, the Chinese SF-6Dv2 value set developed by Wu et al. was used to generate the SF-6Dv2 index score, ranging from − 0.227 (the worst health state) to 1 (full health) [13].

#### KDQOL-36

The KDQOL-36 is a self-report disease-specified HRQOL instrument, which combines the 12-item generic SF-12 instrument together with 24-item kidney disease-specific question, and has been widely used [14]. The SF-12 contains 12 questions covering 8 domains (physical functioning, physical role limitations, bodily pain, general health perceptions, energy/vitality, social functioning, emotional role limitations and mental health), with response levels ranging from 2 to 6 [15]. The 24-item kidney disease-specific questions comprise of three subscales: (1) burden of kidney disease with 4 items, (2) symptoms/problems of kidney disease with 12 items, and (3) effects of kidney disease with 8 items. Each of the 24 items have 5 response levels. For scoring, item responses are transformed linearly onto a 0 to100 scale, with a higher score indicating a better HRQOL [16]. The Chinese version of the KDQOL-36 has been developed and validated among the Chinese patients [17]. The RAND Corporation provided the rules for calculating the KDQOL-36 scores on their website in the form of an Excel file and SAS code [18].

### Statistical analysis

Descriptive statistics were conducted to depict respondents’ characteristics, distribution across levels of the EQ-5D-5L and SF-6Dv2 dimensions and their utility scores. Continuous variables were expressed as means and standard deviations (SD), and categorical variables were shown as frequencies and percentage. The distributions of index scores for both EQ-5D-5L and SF-6Dv2 were plotted in graphs. The mean and median index scores were also reported.

The agreement between EQ-5D-5L and SF-6Dv2 scores was assessed using the intra-class correlation coefficient (ICC). The ICC was calculated using the two-way mixed effects model based on absolute agreement. The ICC ranges from 0 to 1, where a value less than 0.5, between 0.5 and 0.75, and greater than 0.75 indicate poor, moderate, and good agreement, respectively [19]. Bland-Altman plots were generated to visually inspect the utility scores differences between the two instruments. Perfect agreement is indicated when the mean of the between-instrument differences is 0 and they randomly scatter within 1.96 standard deviations around the mean [20].

Given the non-normality of the data, Spearman’s correlation coefficient was used to evaluate the degree of correlation between the EQ-5D-5L and the SF-6Dv2 utility scores, with a coefficient surpassing 0.5 suggesting a strong correlation [21].

The known-groups validity of EQ-5D-5L and SF-6Dv2 utility scores was evaluated based on the assumption that patients in better health would have higher utility scores compared to those in worse health [22, 23]. The study sample was categorized into better and worse health subgroups based on patients’ comorbidities and KDQOL-36 kidney disease-specific scale scores based on mean cut-off. We compared the mean scores differences of the EQ-5D-5L and SF-6Dv2 among the groups known to differ in health. Relative efficiencies (RE) and effect sizes were calculated to assess the sensitivity to known group differences. RE is defined as the ratio of the F-statistic from an ANOVA test comparing utility scores between the better health subgroup and the worse health subgroup [24]. The F-statistic for the EQ-5D-5L index served as the reference value (RE = 1). A higher RE value indicates that the instrument is more likely to detect statistically significant differences between subgroups. Effect sizes were calculated by dividing the between-group difference in the within-group mean by the pooled standard deviation, providing a measure of the magnitude of the difference in utility scores between subgroups [25]. We employed thresholds of 0.2, 0.5, and 0.8 to define small, medium, and large effect sizes, respectively [3–6, 25].

All statistical analyses were performed using STATA (release 15; Stata Corp, College Station, TX, USA) software, with *p* < 0.05 being considered statistically significant.

## Results

### Characteristics of patients

A total of 378 dialysis patients were enrolled in this study, out of which 52.6% was from the WeChat group. Patients’ characteristics are displayed in Table 1. The mean age was 49.1 years (SD, 13.3 years) with approximately equal share of men and women. Although 2/3 have finished at least high school, only 17% are working.

**Table 1:** Characteristics of patients

Characteristic	Total, *n* = 378	
Age (year), mean (SD)	49.1	(13.3)
Sex, n (%)		
Female	191	(50.5)
Male	187	(49.5)
Marital status, n (%)		
Married	266	(70.4)
Single	71	(18.8)
Widowed / Divorced	41	(10.8)
Educational level, n (%)		
No formal / Primary	32	(8.5)
Junior high school	96	(25.4)
High school / technical secondary school	110	(29.1)
Bachelor / College and above	140	(37.0)
Working status, n (%)		
Working	66	(17.5)
Not working	197	(52.1)
Retired	115	(30.4)
Number of comorbidities, n (%)		
<= 2	173	(45.8)
>2	205	(54.2)

*SD* Standard deviation

Table 2 shows the distribution across levels of the EQ-5D-5L and SF-6Dv2 dimensions and descriptive statistics of their utility scores. The EQ-5D-5L score ranged from − 0.391 to 1, with 14.8% of patients reporting full health and 0.5% patients reporting the worst health. The SF-6Dv2 score ranged from − 0.227 to 1, with 1.3% of patients reporting full health and only 1 (0.3%) patient reporting the worst health.

**Table 2:** Distribution across levels of the EQ-5D-5L and SF-6Dv2 dimensions and utility scores

EQ-5D-5L		SF-6Dv2	
Dimensions	Amount	Dimensions	Amount
Mobility		Physical functioning	
Level 1	212(56%)	Level 1	121(32%)
Level 2	75(19.8%)	Level 2	112(29.6%)
Level 3	45(11.9%)	Level 3	71(18.8%)
Level 4	27(7.1%)	Level 4	37(9.8%)
Level 5	19(5.0%)	Level 5	37(9.8%)
Self-care		Role limitation	
Level 1	282(74.6%)	Level 1	131(34.7%)
Level 2	42(11.1%)	Level 2	95(25.1%)
Level 3	23(6.1%)	Level 3	68(18.0%)
Level 4	17(4.5%)	Level 4	42(11.1%)
Level 5	14(3.7%)	Level 5	42(11.1%)
Usual activities		Social functioning	
Level 1	226(59.8%)	Level 1	110(29.1%)
Level 2	78(20.6%)	Level 2	89(23.5%)
Level 3	28(7.4%)	Level 3	83(22.0%)
Level 4	28(7.4%)	Level 4	51(13.5%)
Level 5	18(4.8%)	Level 5	45(11.9%)
Pain/Discomfort		Pain	
Level 1	176(46.6%)	Level 1	113(29.9%)
Level 2	113(29.9%)	Level 2	106(28%)
Level 3	53(14.0%)	Level 3	71(18.8%)
Level 4	27(7.1%)	Level 4	59(15.6%)
Level 5	9(2.4%)	Level 5	24(6.3%)
		Level 6	5(1.3%)
Anxiety/Depression		Mental health	
Level 1	173(45.8%)	Level 1	123(32.5%)
Level 2	108(28.6%)	Level 2	113(29.9%)
Level 3	56(14.8%)	Level 3	68(18.0%)
Level 4	25(6.6%)	Level 4	57(15.1%)
Level 5	16(4.2%)	Level 5	17(4.5%)
		Vitality	
		Level 1	147(38.9%)
		Level 2	96(25.4%)
		Level 3	84(22.2%)
		Level 4	30(7.9%)
		Level 5	21(5.6%)
Utility score		Utility score	
Mean (SD)	0.7169 (0.3403)	Mean	0.5692 (0.2628)
Median	0.8764	Median	0.644
Ceiling effect	56(14.8%)	Ceiling effect	5(1.3%)
Floor effect	2(0.5%)	Floor effect	1(0.3%)

*SD* Standard deviation

**Fig. 1:**
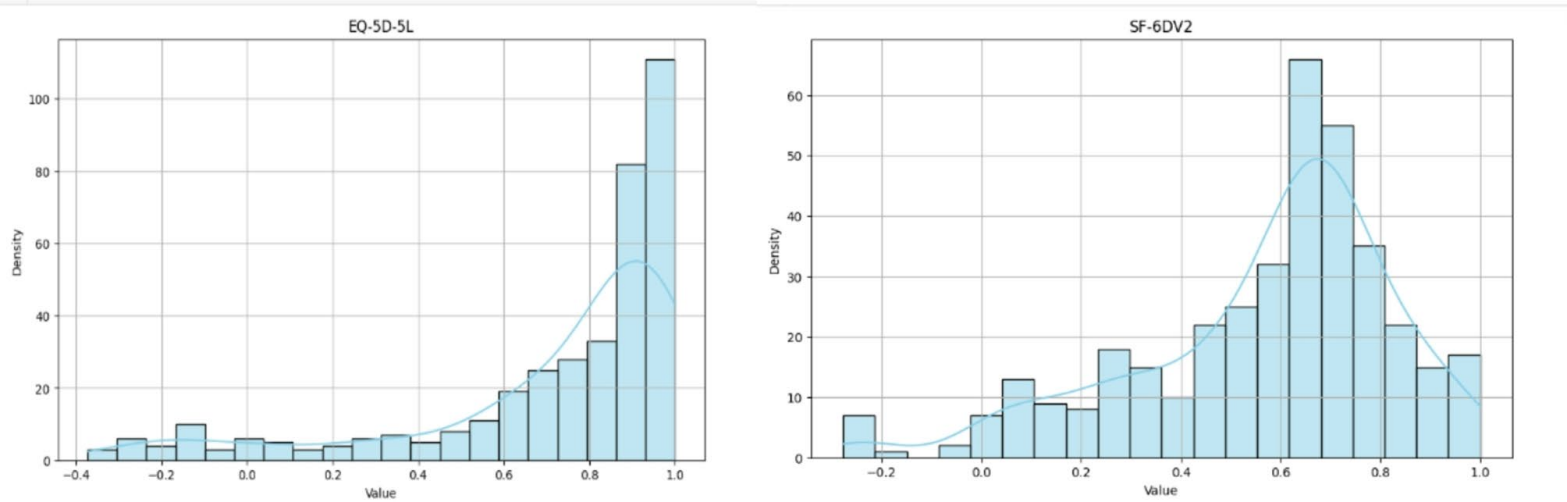
Distribution of EQ-5D-5L and SF-6Dv2 scores

Although both EQ-5D-5L and SF-6Dv2 scores followed a left-skewed distribution (-1.660 and − 0.993), the distribution of EQ-5D-5L scores was more skewed towards perfect health compared to the distribution of SF-6Dv2 and showed significant ceiling effect (Fig. 1).

### Agreement between the EQ-5D-5L and SF-6Dv2 utility scores

**Fig. 2:**
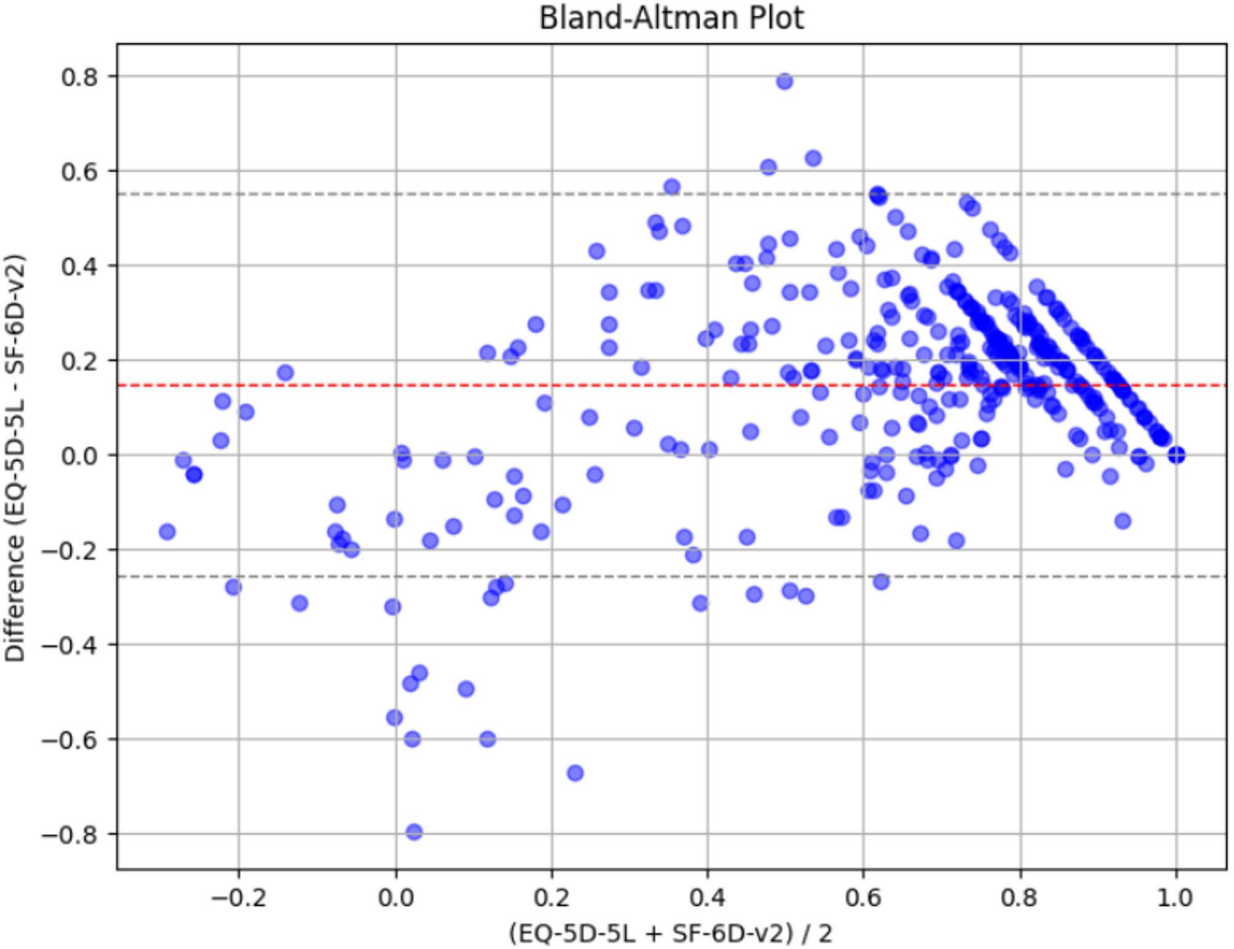
Bland-Altman plot of difference in utility scores between the EQ-5D-5L and the SF-6Dv2

The ICC between the EQ-5D-5L and the SF-6Dv2 utility scores was 0.639 (*p* < 0.001), indicating moderate agreement between the measures. The Bland-Altman plot displayed that the average difference between the two instruments was 0.148 with a standard deviation of 0.205 (Fig. 2). The smaller the value, the smaller the average difference between the two instruments. Figure 2 also shows that the EQ-5D-5L scores were lower than the SF-6Dv2 in patients with lower utility scores.

### Construct validity

The Spearman’s rank correlation coefficient between the EQ-5D-5L and the SF-6Dv2 was 0.767 (*p* < 0.001), indicating a very strong correlation. Patients with more than two comorbidities had lower mean EQ-5D-5L and SF-6Dv2 scores than patients with less comorbidities, statistical significance was achieved in SF-6Dv2 instrument (Table 3). The mean EQ-5D-5L and SF-6Dv2 scores were statistically significantly higher for patients with higher KDQOL-36 kidney disease-specific scale scores (indicating better health status) in all three disease-specific scales (Table 3).

### Sensitivity of the utility scores

Using the EQ-5D-5L index as the reference, the RE values of the SF-6Dv2 were more than 1 for all know group comparisons. The effect sizes reflected a similar trend in the relative sensitivity of the two instruments, with the SF-6Dv2 generally exhibiting larger values compared to the EQ-5D-5L. This suggests that the SF-6Dv2 is more effective at capturing differences between the better health subgroup and the worse health subgroup than the EQ-5D-5L. The SF-6Dv2 showed greater differences in utility between the known groups than the EQ-5D-5L.

**Table 3:** Known group validity and sensitivity of EQ-5D-5L and SF-6Dv2

Grouping variable	*N*	Mean (SD)	
		EQ-5D-5L	SF-6Dv2
Number of comorbidities			
<= 2	173	0.73 (0.33)	0.61 (0.25)
>2	205	0.69 (0.35)	0.53 (0.27)
Mean difference		0.04	0.08**
RE		1	8.32
Effect size		0.11	0.33
Disease-targeted scales of KDQOL-36^&^			
Symptoms			
<= 73	192	0.58 (0.38)	0.44 (0.26)
>73	186	0.85 (0.22)	0.70 (0.18)
Mean difference		0.27***	0.26***
RE		1	1.86
Effect size		0.87	1.19
Effects			
<= 56	188	0.58 (0.39)	0.43 (0.26)
>56	190	0.85 (0.21)	0.71 (0.17)
Mean difference		0.26***	0.28***
RE		1	2.23
Effect size		0.84	1.26
Burden			
<= 19	191	0.60 (0.39)	0.45 (0.27)
>19	187	0.83 (0.23)	0.69 (0.19)
Mean difference		0.23***	0.23***
RE		1	1.76
Effect size		0.73	1.00

*SD* Standard deviation, *RE* Relative efficiency

* *p* < 0.05, ** *p* < 0.01, *** *p* < 0.001 (t test of the difference between known groups)

^&^ Cut-off values are median scores

## Discussion

Our study found that the EQ-5D-5L and the SF-6Dv2 instruments were both valid for dialysis patients and the SF-6Dv2 instrument was more sensitive compared to the EQ-5D-5L. The two instruments gave different estimates for utility differences between groups of patients and were not interchangeable. These findings highlighted the importance of in-depth investigation of different generic HRQOL instruments in the outcomes research of ESRD area.

Correlation between the EQ-5D-5L and the SF-6Dv2 was strong (≥ 0.5), consistent with that found in previous studies [7, 26]. Agreement between the EQ-5D-5L and the SF-6Dv2 scores was moderate in our study while another study in Singapore found that the agreement between the EQ-5D-5L and the SF-6D scores was poor [7]. Although the degree of agreement was different between our study and the Singapore study, both studies found the EQ-5D-5L tend to derive lower scores than the SF-6D/SF-6Dv2 for patients with lower health, indicating that the two instruments cannot be used interchangeably.

Both instruments demonstrated know groups validity as the mean utility scores showed expected difference between subgroups of patients with better and worse health state. The SF-6Dv2 was more sensitive in discriminating patients with different levels of comorbidity conditions and KDQOL-36 kidney disease-specific scales.

Regarding the comorbidity conditions, another study found that the EQ-5D-5L was superior sensitive to the SF-6D [7]. The reasons for the inconsistent results may include the source of the SF-6D scores, and the definition of comorbidity conditions between studies. However, our results were consistent with findings from the general population, hearing impaired adults and liver transplant patients [22, 26–28]. All these findings indicated that the sensitivity of the two generic instruments may be population-specific.

Regarding the KDQOL-36 kidney disease-specific scales, our results on the sensitivity of the instruments were consistent with a previous study [7]. A possible reason for the greater sensitivity of SF-6Dv2 on kidney disease-specific scales could be that the SF-6Dv2 assess the health status based on the “last four weeks” whereas the EQ-5D-5L based on the survey day (“today”). A longer recall period may offer more stable reflection of the actual situation, rather than being affected by chance.

The EQ-5D-5L exhibited greater utility gains than the SF-6Dv2 in our study, which was consistent with findings from previous studies [3–6, 29]. These results indicated that using the EQ-5D-5L in cost-utility analysis may lead to overestimate of incremental effect and further incremental cost-effectiveness ratios and favor chances of adopting more expensive but more effective alternatives. Therefore, policy decision making based on economic evaluation should carefully consider the utility measurement instruments.

The SF-6Dv2 showed the greater difference in utility scores between the known groups compared to the EQ-5D-5L in our study. In contrast, the EQ-5D-5L in our study exhibited a notable ceiling effect, suggesting that SF-6Dv2 may offer greater sensitivity in capturing variations in HRQOL among Chinese dialysis patients. This difference may be due to the broader range of dimensions measured by the SF-6Dv2 compared to the EQ-5D-5L. In addition to physical and mental health, it also captures vitality and social functioning, allowing it to detect more subtle changes in health status. Moreover, a longer recall period (4-weeks vs. one-day) may also lead individuals to report more severe symptoms and lower utility scores [30]. Another possible reason is that the wording of the SF-6Dv2 may make Chinese dialysis respondents less likely to report the highest possible score, which is reflected in the lower ceiling effects observed across each dimension. As a result, the EQ-5D-5L may fail to detect subtle impairments in quality of life, making the SF-6Dv2 a more suitable instrument for assessing dialysis patients with milder symptoms.

The findings were inconsistent with the Singapore study which found that the EQ-5D-5L showed greater difference in utility scores between the known groups except for the KDQOL-36 burden scale [7]. The possible reasons may be due to the source of the SF-6D scores (SF-12 based SF-6D scores in the Singapore study) and different culture context. Two additional studies conducted in China—one among patients with hemophilia and the other among survivors of classical Hodgkin lymphoma (CHL)—also found that the EQ-5D-5L demonstrated better discriminatory power than the SF-6Dv2 [31, 32]. In contrast to our findings, this highlights the heterogeneity across different patient populations. Moreover, the CHL study reported a ceiling effect for EQ-5D-5L and suggested that variations in measurement properties of utility instruments may exist across cultural contexts [32]. Another study focusing on Chinese patients with spinal and bulbar muscular atrophy identified a substantial floor effect in the SF-6D, potentially leading to an underestimation of utility scores [33]. These findings collectively underscore the need to select appropriate instruments for different chronic health conditions. SF-6D may be more suitable for distinguishing among patients with milder symptoms, whereas EQ-5D-5L appears to perform better in detecting differences in more severe cases [33].

There are several advantages of this study. First, we included patients from different hospitals in China, which can be considered representative of Chinese dialysis patients. Second, as far as we know, there have been no studies exploring the choice of EQ-5D-5L and SF-6Dv2 when estimating the utility for Chinese dialysis patients. In addition, we used comprehensive and advanced methods to systematically compare the two generic utility measurement instruments.

In addition to the advantages, there are some limitations worth noting. First, the cross-sectional design lacks the ability to assess the longitudinal sensitivity of the two instruments when the health state of patients changed. Future study should therefore consider a longitudinal design to evaluate how well each instrument captures within-subject changes in health status. Second, the presence of numerous missing values in clinical data of our dataset limited our ability to fully assess the known groups’ validity and sensitivity of the two instruments. For instance, patients with varying blood hemoglobin levels and dialysis adequacy exhibited different utility scores, which were not examined in our study. Third, the use of hospital-based recruitment in major cities and WeChat group dissemination may introduce selection bias. Participants were likely to be more urban, digitally literate, and connected to nephrology networks than the general dialysis population. This may limit the generalizability of the findings to less connected or rural patients.

## Conclusion

Our study suggested that both the EQ-5D-5L and SF-6Dv2 are valid and sensitive when assessing the utility scores of Chinese dialysis patients. However, the SF-6Dv2 showed higher sensitivity compared to the EQ-5D-5L, and they are not interchangeable. The SF-6Dv2 is more appropriate to measure the utility scores of Chinese dialysis patients.

## Data Availability

The data that support the findings of this study are available from the corresponding author upon reasonable request.
